# Primary Native Bile Culture Patterns Collected During ERCP: Microbial Profiles in Malignant Versus Benign Biliary Obstruction

**DOI:** 10.3390/microorganisms14102128

**Published:** 2026-09-22

**Authors:** Szu-Yao Wang, Chun Yang, Chun-Fang Tung, Yi-Jun Liao, Chia-Chang Chen, Yen-Chun Peng

**Affiliations:** 1 Division of Gastroenterology, Department of Internal Medicine, Taichung Veterans General Hospital, Taichung 407219, Taiwan; a120819ck@gmail.com (S.-Y.W.); yangchun860815@gmail.com (C.Y.); et@vghtc.gov.tw (C.-F.T.); s19001029@gmail.com (Y.-J.L.); jcchen@vghtc.gov.tw (C.-C.C.); 2 School of Medicine, National Yang Ming Chiao Tung University, Taipei 112304, Taiwan; 3 Department of Post-Baccalaureate Medicine, College of Medicine, National Chung Hsing University, Taichung 402202, Taiwan

**Keywords:** bile culture, ERCP, biliary obstruction, microbiota, benign disease, malignancy

## Abstract

Acute biliary tract infection is a serious complication of biliary obstruction, and understanding the native biliary microbiota is essential for optimizing empirical antimicrobial therapy. We retrospectively reviewed 404 patients who underwent their first endoscopic retrograde cholangiopancreatography (ERCP) for biliary obstruction between January and December 2024; bile was aspirated before any therapeutic manipulation, and microbiological characteristics were compared between benign and malignant obstruction. Among 404 patients, 365 bile samples were collected, and overall culture positivity was 68.8%. Benign obstruction demonstrated significantly higher positivity than malignant obstruction (71.9% vs. 59.3%, *p* = 0.04). Monomicrobial Gram-positive organisms were disproportionately more common in benign obstruction (17.2% vs. 7.7%, *p* = 0.03), whereas monomicrobial fungal organisms were more frequently isolated in malignant obstruction (9.9% vs. 4.4%, *p* = 0.07). Polymicrobial infections occurred in approximately one-quarter of cases, and anaerobes were relatively uncommon (7.9%). Bile cultures obtained during the initial ERCP revealed distinct microbial profiles associated with different etiologies of biliary obstruction, suggesting that the distribution of identified organisms varies according to the underlying cause of obstruction.

## 1. Introduction

Acute biliary tract infection, or cholangitis, is usually caused by biliary obstruction of benign or malignant origin and is a potentially life-threatening condition that requires prompt recognition and management [1]. The ASGE guidelines for acute cholangitis recommend prompt diagnosis and early intervention with antibiotics and biliary decompression [2,3]. An international guideline on source control in emergency general surgery further states that biliary obstruction and stasis constitute an anatomic “source” of infection, requiring physical intervention—typically timely drainage or decompression—to achieve effective resolution of biliary sepsis [4]. Without timely intervention, including biliary drainage, mortality rates remain unacceptably high; in critically ill patients, early biliary drainage within 24 h has been associated with reduced rates of persistent organ failure and shorter hospital stay [3,5]. The most commonly used drainage methods are endoscopic retrograde cholangiopancreatography (ERCP), percutaneous transhepatic cholangiodrainage (PTCD), and endoscopic ultrasonography-guided internal drainage [2,6,7]. In addition to drainage, antimicrobial therapy is a cornerstone of management. Initial empirical therapy is reasonable; however, adjustment according to microbiological findings from bile is mandatory for optimizing outcomes [3,8,9,10]. The updated Clinical Practice Guideline issued by the Infectious Diseases Society of America states that bile culture is a critical diagnostic modality in the management of cholangitis, enabling direct identification of causative pathogens and determination of antimicrobial susceptibilities [9]. Bile aspiration obtained during ERCP provides valuable information that complements blood cultures, with a significantly higher diagnostic yield, and enables targeted antibiotic therapy [6,8,10,11]. Recent data suggest that antimicrobial regimens guided by bile culture results achieve > 80% effective coverage and may be a powerful tool for clinical decision-making regarding antibiotic use [12].

Most previous studies of bile culture have included samples from patients who underwent prior ERCP interventions, such as endoscopic sphincterotomy, balloon dilation, or biliary drainage. However, these procedures carry a risk of disrupting the biological barrier of the biliary tract and the bowel microbiome, altering the relationship between the biliary and bowel microbiota. We therefore collected bile during ERCP from patients who had never received any prior treatment, focusing on culture results from native bile. We hypothesized that the underlying etiology of obstruction shapes the biliary microbial environment, influencing both the clinical course and the microbiological spectrum.

Herein, we conducted a retrospective study using bile samples collected from patients undergoing ERCP with common bile duct cannulation, ensuring that none of the patients had a prior history of ERCP treatment. This approach enabled us to obtain native bile, offering a more accurate representation of the microbial environment of the bile duct without the influence of previous interventions, and to minimize potential alterations in bile composition that could result from prior sphincterotomy or balloon dilation.

## 2. Materials and Methods

### 2.1. Study Design and Patient Population

This was a single-center, retrospective cohort study conducted at Taichung Veterans General Hospital (VGHTC), a medical center in Taichung, Taiwan. Adult patients (≥18 years) who underwent their first ERCP for biliary obstruction between 1 January 2024 and 31 December 2024 were eligible for inclusion. First-ERCP status was verified using referral records and patient claims history to identify any prior ERCP performed at other institutions. Patients with prior ERCP, endoscopic sphincterotomy, biliary stent placement, or percutaneous biliary drainage were excluded to minimize alterations in the biliary microbiota caused by previous interventions. Patients with incomplete medical records or unsuccessful bile aspiration were also excluded. Patients were categorized into two groups: those with malignancy-related acute biliary obstruction and those with benign causes of obstruction, including choledocholithiasis. This study was approved by the Institutional Review Board of Taichung Veterans General Hospital (protocol code CE25374C; approved 30 June 2025), and the requirement for informed consent was waived owing to the retrospective nature of this study.

### 2.2. Data Collection

Demographic data, clinical characteristics, comorbidities, laboratory parameters, and imaging findings were obtained from electronic medical records. Etiologies of obstruction were classified as benign (e.g., choledocholithiasis, benign stricture) or malignant (e.g., pancreatic cancer, cholangiocarcinoma, metastatic disease). Biochemical profiles collected included liver function tests (aspartate aminotransferase [AST], alanine aminotransferase [ALT], alkaline phosphatase [ALP], total and direct bilirubin) and inflammatory markers (C-reactive protein [CRP], white blood cell count).

### 2.3. ERCP Procedure and Bile Culture Collection

All ERCP procedures were performed by 4 experienced endoscopists. During ERCP, bile specimens were aseptically aspirated from the common bile duct using a sterilized catheter immediately after successful biliary cannulation. To minimize contamination, samples were obtained prior to instillation of any contrast medium, saline flushing, or therapeutic manipulation. Approximately 2–5 mL of bile was collected into sterile containers under direct endoscopic guidance to ensure accuracy and avoid mixture with duodenal or pancreatic secretions. Bile samples were promptly transported under controlled conditions to the microbiology laboratory, typically within 1 h of retrieval, and processed without delay. For microbiological culture, bile samples were inoculated onto standard aerobic and anaerobic culture media, including 5% sheep blood agar, MacConkey agar, and anaerobic blood agar plates. Inoculated plates were incubated under appropriate atmospheric conditions (aerobic at 35–37 °C and anaerobic using an anaerobic chamber) to allow optimal growth of both aerobic and anaerobic organisms.

### 2.4. Microbial Identification

Bile and blood specimens were cultured using standard aerobic and anaerobic techniques. Identification of bacterial and fungal isolates was performed with automated systems (VITEK 2, bioMérieux, Marcy-l’Étoile, France) and confirmed by conventional biochemical assays where necessary. Isolates were categorized as Gram-negative, Gram-positive, fungal, or anaerobic organisms. Cases with more than one organism isolated from a single specimen were defined as polymicrobial infections.

### 2.5. Statistical Analysis

Continuous variables were expressed as medians and interquartile ranges (IQR) and compared using the Mann–Whitney U test. Categorical variables were reported as counts and percentages and compared using the chi-square or Fisher’s exact test, as appropriate. To contextualize the magnitude of between-group differences, standardized mean differences (SMD) with 95% confidence intervals (CI) were additionally calculated for all comparative variables, as detailed in the footnotes of Table 1. A *p* value < 0.05 was considered statistically significant. All analyses were performed using SPSS version 27.0 (IBM Corp., Armonk, NY, USA). No generative artificial intelligence tools were used to design this study, collect data, or perform the statistical analyses reported herein; generative AI assistance was used only for language editing and reference/table formatting of the manuscript text, as detailed in the Acknowledgments.

### 2.6. Ethical Considerations

The requirement for informed consent was waived owing to the retrospective nature of this study, and all data were anonymized to protect patient confidentiality. This study adhered to the principles of the Declaration of Helsinki and was approved by the Institutional Review Board of Taichung Veterans General Hospital (CE25374C).

## 3. Results

### 3.1. Baseline Characteristics

A total of 404 patients who underwent their first ERCP at our hospital between 1 January 2024 and 31 December 2024 were enrolled, including 304 with benign obstruction and 100 with malignant obstruction; baseline characteristics are summarized in Table 1. Overall, 182 patients (45%) were female, with comparable proportions in the benign (134/304, 44%) and malignant (48/100, 48%) obstruction groups (*p* = 0.54). Diabetes mellitus was present in 99 patients (25%) overall, with similar proportions in the benign (74/304, 24%) and malignant (25/100, 25%) groups (*p* = 0.87). The median age was 66 years (IQR, 55.8–74) overall and was similar between the benign (65.5 years; IQR, 54.8–75) and malignant (66 years; IQR, 57–73) obstruction groups (*p* = 0.78).

Biochemical profiles, however, demonstrated marked between-group differences. Patients with malignant obstruction had substantially higher alkaline phosphatase levels than those with benign obstruction (median, 386.5 U/L; IQR, 235–532.8 vs. 145.5 U/L; IQR, 101–240.3; *p* < 0.001). Similarly, total bilirubin was significantly higher in the malignant group (median, 6.97 mg/dL; IQR, 3.55–11.46 vs. 2.0 mg/dL; IQR, 0.90–4.36; *p* < 0.001), as was direct bilirubin (median, 5.19 mg/dL; IQR, 2.31–8.18 vs. 1.14 mg/dL; IQR, 0.41–2.98; *p* < 0.001).

In contrast, serum transaminase levels did not differ significantly between groups. The median AST level was 96.5 U/L (IQR, 37–257.5) in the benign group and 119 U/L (IQR, 73.8–212.3) in the malignant group (*p* = 0.27), while the median ALT level was 140 U/L (IQR, 38.8–290) and 118 U/L (IQR, 60.5–257), respectively (*p* = 0.36). CRP levels were numerically higher in patients with malignant obstruction (median, 2.13 mg/dL; IQR, 1.05–6.20) than in those with benign obstruction (median, 1.39 mg/dL; IQR, 0.34–6.74), but this difference was not statistically significant (*p* = 0.15).

### 3.2. Bile Culture Positivity by Obstruction Etiology

Bile samples were successfully obtained in 365 of 404 examinations (Figure 1). Bile culture results were available for 274 patients in the benign group and 91 patients in the malignant group. Among these, 114 samples showed no microbial growth, whereas 251 demonstrated positive bile cultures, yielding an overall positivity rate of 68.8% (251/365). The indications for ERCP, together with the corresponding numbers of bile and blood cultures collected, are summarized in Table 2.

### 3.3. Microbial Spectrum in Benign Versus Malignant Obstruction

The bile culture collection rate was comparable between groups (90% [274/304] in the benign group vs. 91% [91/100] in the malignant group; *p* = 0.81). Among cases with bile cultures performed (benign, *n* = 274; malignant, *n* = 91), culture positivity was significantly higher in the benign group than in the malignant group (71.9% [197/274] vs. 59.3% [54/91]; *p* = 0.04; risk difference, 12.6%, 95% CI 1.1% to 24.0%). Polymicrobial growth was observed in 25.9% (71/274) of benign cases and 20.9% (19/91) of malignant cases, with no significant between-group difference (*p* = 0.34; risk difference, 5.0%, 95% CI −4.8% to 14.9%).

Among the cases with bile cultures performed, monomicrobial Gram-positive organisms were more frequently identified in the benign group than in the malignant group (17.2% [47/274] vs. 7.7% [7/91]; *p* = 0.03; risk difference, 9.5%, 95% CI 2.4% to 16.5%). Monomicrobial Gram-negative organisms were identified in 24.5% (67/274) of benign cases and 20.9% (19/91) of malignant cases, with no significant difference between groups (*p* = 0.52; risk difference, 3.6%, 95% CI −6.2% to 13.4%). Monomicrobial *Candida* was identified in 4.4% (12/274) of benign cases and 9.9% (9/91) of malignant cases. Although the proportion was numerically higher in the malignant group, the difference was not statistically significant (*p* = 0.07; risk difference, −5.5%, 95% CI −12.1% to 1.1%). These findings are summarized in Table 3 and illustrated in Figure 2 and Figure 3.

### 3.4. Distribution of Isolated Organisms

A total of 339 microorganisms were isolated from bile cultures. Gram-negative bacteria constituted the majority of isolates (211, 62.2%), followed by Gram-positive bacteria (96, 28.3%) and fungi (32, 9.4%). Among Gram-positive organisms, *Enterococcus* species were the most frequently identified (63, 18.6%), followed by *Streptococcus* species (15, 4.4%), *Staphylococcus* species (11, 3.2%), and other genera (7, 2.1%). The predominant Gram-negative isolates included *Escherichia coli* (65, 19.2%), *Klebsiella* species (45, 13.3%), *Enterobacter cloacae* (24, 7.1%), *Pseudomonas* species (22, 6.5%), *Citrobacter* species (5, 1.5%), and other less common isolates (50, 14.7%). Fungal isolates accounted for 9.4% of all organisms, of which *Candida albicans* was most common (29, 8.6%), followed by other *Candida* species (3, 0.9%). Anaerobic isolates were relatively uncommon, accounting for only 7.9% of all organisms identified (Table 4). Importantly, these anaerobic isolates were already included within the Gram-positive and Gram-negative bacterial categories and therefore should not be counted as an additional category or added separately to the overall isolate count. These findings indicate a predominance of Gram-negative bacteria, particularly Enterobacteriaceae, among patients with biliary tract infections.

## 4. Discussion

In our study, bile cultures yielded positive results in 68.8% of 365 patients. Patients with benign biliary obstruction exhibited significantly higher culture positivity than those with malignant obstruction (71.9% vs. 59.3%, *p* = 0.04; risk difference, 12.6%, 95% CI 1.1% to 24.0%). Gram-positive organisms were significantly more prevalent in patients with benign obstruction (17.2% vs. 7.7%, *p* = 0.03; 9.5% (2.4% to 16.5%), consistent with their recognized role in ascending cholangitis and post-ERCP infections. Interestingly, fungal isolates, predominantly *Candida* spp., were more frequently detected in the malignant obstruction group (9.9% vs. 4.4%), although this trend did not reach statistical significance (*p* = 0.07; −5.5% (−12.1% to 1.1%). The higher fungal yield in malignancy may reflect prolonged biliary infection or immunocompromised states. Moreover, polymicrobial growth was observed in approximately one-quarter of cases, underscoring the polymicrobial nature of biliary infections, while anaerobic organisms remained relatively uncommon (7.9%), aligning with prior reports that highlight their limited role in biliary sepsis.

Bile within the intrahepatic and extrahepatic biliary tree has traditionally been considered sterile under physiological conditions, a concept attributed to the protective function of the sphincter of Oddi and the antegrade flow of bile [13]. This sterility can be disrupted by interventions that involve the papilla, which serves as the biological barrier of the duodenum. Subsequent studies demonstrated that biliary instrumentation, stenting, and obstruction alter this environment, leading to microbial colonization and infection; enterococcal and polymicrobial infections are more common in patients with prior biliary stents [14]. A German study analyzing 289 bile cultures reported positivity rates of up to 85% in patients undergoing ERCP, further highlighting the increased risk of fungal and multidrug-resistant organisms in previously instrumented patients [11]. Stathopoulos et al. conducted a large retrospective analysis of 405 ERCP-obtained bile cultures in acute cholangitis and reported an 86.7% positivity rate; prior sphincterotomy and biliary stenting were strong independent predictors of multidrug-resistant organisms and fungal isolation, illustrating the profound impact of instrumentation on biliary microbiology [15]. The interpretation of bile culture data is therefore strongly influenced by whether aspirates are obtained from native bile ducts or from patients with prior biliary instrumentation. In native bile samples, such as those obtained during first-time ERCP without prior treatment, the microbiological profile more closely reflects the true pathogen spectrum of biliary obstruction; in contrast, bile collected from patients with indwelling stents or after multiple ERCP procedures is more likely to demonstrate polymicrobial growth, colonization by enteric flora, and multidrug-resistant organisms owing to selective pressure and biofilm formation.

The distinction in bile culture positivity rates between native bile and bile obtained after prior biliary instrumentation is clinically meaningful. As demonstrated by Gromski et al., the bile culture positivity rate reached 91.8% in their overall cohort but declined to 86.1% when limited to patients without prior stent placement, highlighting how instrumentation alters both the culture yield and the spectrum of microorganisms [7]. Krupik et al., in a prospective single-center study of patients with mild-to-moderate ascending cholangitis, reported a positivity rate of 66.2% in native bile aspirates [16]. These findings indicate that bile obtained from first-time ERCP procedures provides a more accurate representation of the baseline microbial environment than bile from previously manipulated ducts. Our study focused on first-time ERCP cases, ensuring that only native bile was analyzed; by excluding patients with prior biliary stenting or repeated instrumentation, our data reflect the intrinsic microbiological landscape of benign and malignant obstruction. The observed positivity rate of 68.8% therefore aligns closely with prior native-bile studies, while remaining lower than rates reported in previously stented populations.

Importantly, a positive bile culture should not be interpreted as definitive evidence of active clinical infection. Culture positivity indicates the presence of detectable microorganisms in the biliary tract at the time of sampling, but does not establish that the isolated organism was responsible for clinical infection in an individual patient. The interpretation of bile culture results should therefore be considered in conjunction with clinical and radiological findings, including fever, leukocytosis, imaging evidence of infection, and clinical response to antimicrobial therapy. In the present study, these clinical parameters were not systematically incorporated into the microbiological analysis. Accordingly, the differences in bile culture positivity and microbial profiles observed between benign and malignant biliary obstruction should be interpreted as differences in biliary microbial detection rather than direct evidence of differences in active biliary infection.

In our study, Gram-negative Enterobacteriaceae—particularly *Escherichia coli* and *Klebsiella* species—predominated in positive bile cultures, consistent with prior reports from other centers. In a study of patients with biliary tract disease, Liu et al. found that *E. coli* and *K. pneumoniae* accounted for 40.5% and 15.8% of isolates, respectively, confirming the dominant role of Enterobacteriaceae in biliary infections [17]. The predominance of these Gram-negative organisms further supports the concept that duodenal–biliary reflux is a key route for microbial invasion; indeed, Han et al. demonstrated that biliary microbiota in choledocholithiasis patients closely resemble duodenal microbiota, supporting the hypothesis of retrograde bacterial translocation from the gut into the biliary tree [18]. A literature review encompassing nine studies with a combined total of 2175 patients revealed 643 with documented causes of biliary obstruction; the reported bile culture positivity rate across these studies was approximately 68%, with *E. coli*, *Klebsiella*, and *Enterobacter* consistently ranked among the most frequent isolates. These findings align with the pathophysiological model in which bile stasis and increased intraductal pressure facilitate retrograde ascent of enteric bacteria into the biliary system [19]. Taken together, our findings and the existing literature converge on a model in which biliary obstruction disrupts biliary defense mechanisms, elevates intraductal pressure, and permits duodenal bacteria, especially Enterobacteriaceae, to reflux and colonize the biliary system.

Differences in bile culture between benign and malignant obstruction could potentially be used to infer the duration of obstruction. Bile culture positivity was significantly higher in benign than in malignant obstruction (71.9% [197/274] vs. 59.3% [54/91], *p* = 0.04). A previous retrospective study of 262 patients (124 with malignant and 138 with benign obstruction) that analyzed the bacterial species involved in acute cholangitis and their antimicrobial resistance patterns similarly observed a higher culture positivity rate in benign than malignant etiologies (55.7% vs. 44.3%) [20]. One possible, though unmeasured, explanation for this difference is that benign obstruction, particularly that related to choledocholithiasis, may be intermittent or recurrent and could therefore promote periods of bile stasis that facilitate bacterial colonization or infection. In contrast, malignant obstruction often develops as a more progressive and persistent narrowing. However, obstruction duration and the temporal pattern of obstruction were not directly assessed in our study. Therefore, this proposed relationship should be regarded as a speculative explanation rather than evidence that culture positivity reflects or indicates the duration of biliary obstruction [21].

With respect to the spectrum of microorganisms, our findings demonstrate that Gram-positive isolates were significantly enriched in patients with benign obstruction. This observation aligns with prior studies reporting a predominance of *Enterococcus* in bile cultures from patients with non-malignant etiologies [20]. Similarly, Krupik et al. (2025) demonstrated that *Enterococcus* spp. were the most prevalent organisms in patients with acute cholangitis and a naïve papilla, accounting for more than half of all isolates [16]. In contrast, fungal organisms, predominantly *Candida albicans*, were more frequently identified in patients with malignant obstruction than those with benign disease (9.9% vs. 4.4%), consistent with previous studies reporting an increased prevalence of *Candida* species in bile cultures from patients with malignancy or immunocompromised states [8]. Lenz et al. (2009, 2014) also demonstrated that *Candida* species were commonly detected in bile samples obtained during endoscopic retrograde cholangiography, particularly among individuals with malignant biliary obstruction or prior biliary instrumentation [22,23]. Similarly, Hartmann et al. (2023) noted the emerging recognition of biliary fungal microbiota as a contributor to adverse outcomes in hepatobiliary disease [24]. The higher fungal yield in malignant obstruction may reflect impaired local immunity, prolonged biliary stasis, and repeated endoscopic interventions, which collectively create an environment conducive to fungal colonization and biofilm formation. These findings underscore the importance of considering fungal pathogens in patients with malignant biliary disease, particularly when infection persists despite adequate antibacterial therapy.

Polymicrobial infections were detected in approximately one-quarter of cases in our cohort, consistent with previous studies reporting rates of 25–30%, and even higher in certain populations [20,25]. Anaerobic isolates were rare in our series (27/339, 7.9%), in line with previous culture-based investigations; Lavillegrand et al. (2021) analyzed a multicenter ICU cohort and reported that anaerobes accounted for 5% of all positive bile cultures [1]. Nevertheless, next-generation sequencing (NGS) consistently detects additional microbial taxa, including anaerobic species, that are missed by culture-based methods, suggesting that the true diversity and contribution of anaerobes within the biliary microbiota are likely underestimated [24,26].

This study had several key strengths, including a large sample size, systematic bile culture collection at first ERCP, and strict exclusion of patients with prior interventions, ensuring that results reflected native bile microbiota. However, several limitations should be noted. First, the retrospective design may have introduced selection bias and residual confounding, and some clinical information, particularly prior antibiotic exposure, may not have been consistently documented. Previous antibiotic use could have reduced culture yield and potentially influenced the observed differences in microbial profiles between benign and malignant obstruction. Second, this study was conducted at a single institution, which may limit generalizability; variations in local microbial flora, treatment protocols, and patient demographics may affect applicability to broader populations, and multicenter studies would be necessary to validate these results across different settings. Third, although ERCP was performed by experienced gastroenterologists, variability in ERCP technique and bile-sample handling among practitioners could have introduced variability in the microbial cultures obtained; factors such as timing of bile collection, choice of culture media, and procedural variation may have affected the identification and quantification of microbial organisms, potentially confounding this study’s outcomes. Finally, our microbiological assessment relied on culture-based methods, with limited fungal-selective and anaerobic culture conditions. Culture-based detection may therefore underestimate fastidious, anaerobic, fungal, or low-biomass organisms compared with molecular-based methods. These limitations should be considered when interpreting the observed microbial differences.

In future research, we suggest further characterization of the antimicrobial resistance profiles of biliary isolates to determine whether resistance patterns differ between benign and malignant obstruction; such analyses could help refine empirical antibiotic strategies and improve infection control in patients with obstructive jaundice. Expanding this work to include bile microbiota sequencing would provide valuable insights into how the biliary microbial community influences infection dynamics and possibly cancer pathophysiology. Previous studies have demonstrated distinct alterations in the bile microbiome between benign and malignant pancreaticobiliary diseases [27,28], and significant microbial differences among various types of pancreaticobiliary cancers have also been reported [29,30]. Collectively, these findings indicate that specific microbial signatures correlate with disease phenotypes and may reflect underlying biological mechanisms [31]. Advanced molecular techniques, such as next-generation sequencing, could offer a more comprehensive characterization of the bile microbiome [32,33]. A multicenter design incorporating diverse patient populations would enhance statistical power, validate our findings, and facilitate translational applications in antimicrobial stewardship and personalized therapy for acute biliary infections.

## 5. Conclusions

Benign and malignant biliary obstruction showed distinct bile microbiological profiles. Benign obstruction was associated with higher culture positivity and greater Gram-positive enrichment, whereas malignant obstruction showed a trend toward increased fungal colonization. A key strength of this study was the inclusion of patients undergoing first-time ERCP without prior biliary instrumentation, allowing characterization of native bile microbiology in a treatment-naïve setting. These findings suggest that obstruction etiology and local microbiological patterns may be relevant when considering empiric therapy. However, the results should be interpreted cautiously given the single-center, retrospective, exploratory design and the absence of antimicrobial susceptibility and multivariable analyses.

## Figures and Tables

**Figure 1:**
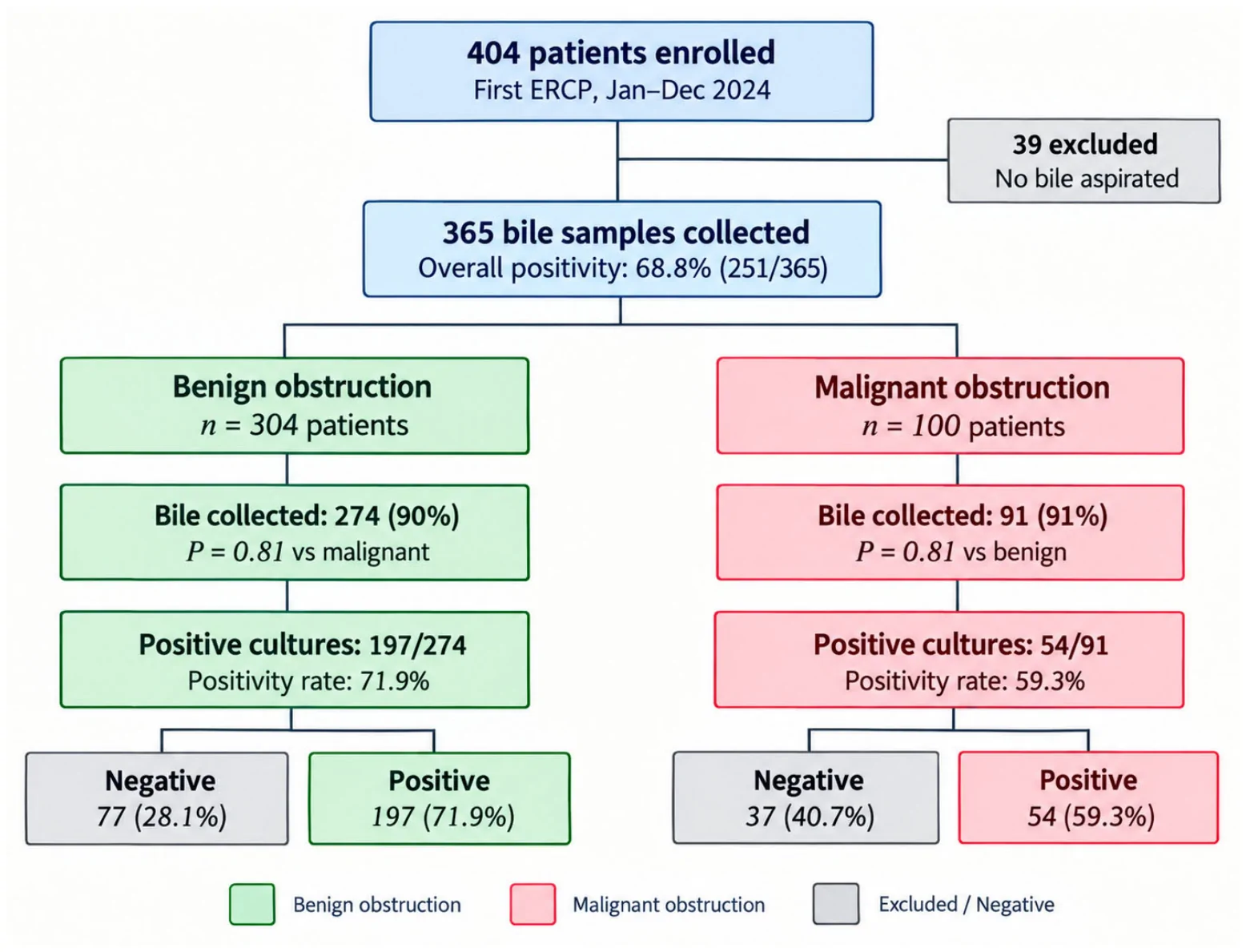
Flow diagram of patient enrollment and bile culture results. Among 404 patients undergoing their first ERCP in 2024, 39 were excluded because no bile could be aspirated, leaving 365 bile samples collected (overall culture positivity 68.8%, 251/365). Patients were stratified into a benign obstruction group (*n* = 304) and a malignant obstruction group (*n* = 100). Bile collection rates were comparable between groups (274/304 [90%] vs. 91/100 [91%], *p* = 0.81; SMD −0.03). Among samples collected, the benign group demonstrated a significantly higher culture-positivity rate than the malignant group (197/274 [71.9%] vs. 54/91 [59.3%], *p* = 0.04; SMD 0.27, small).

**Figure 2:**
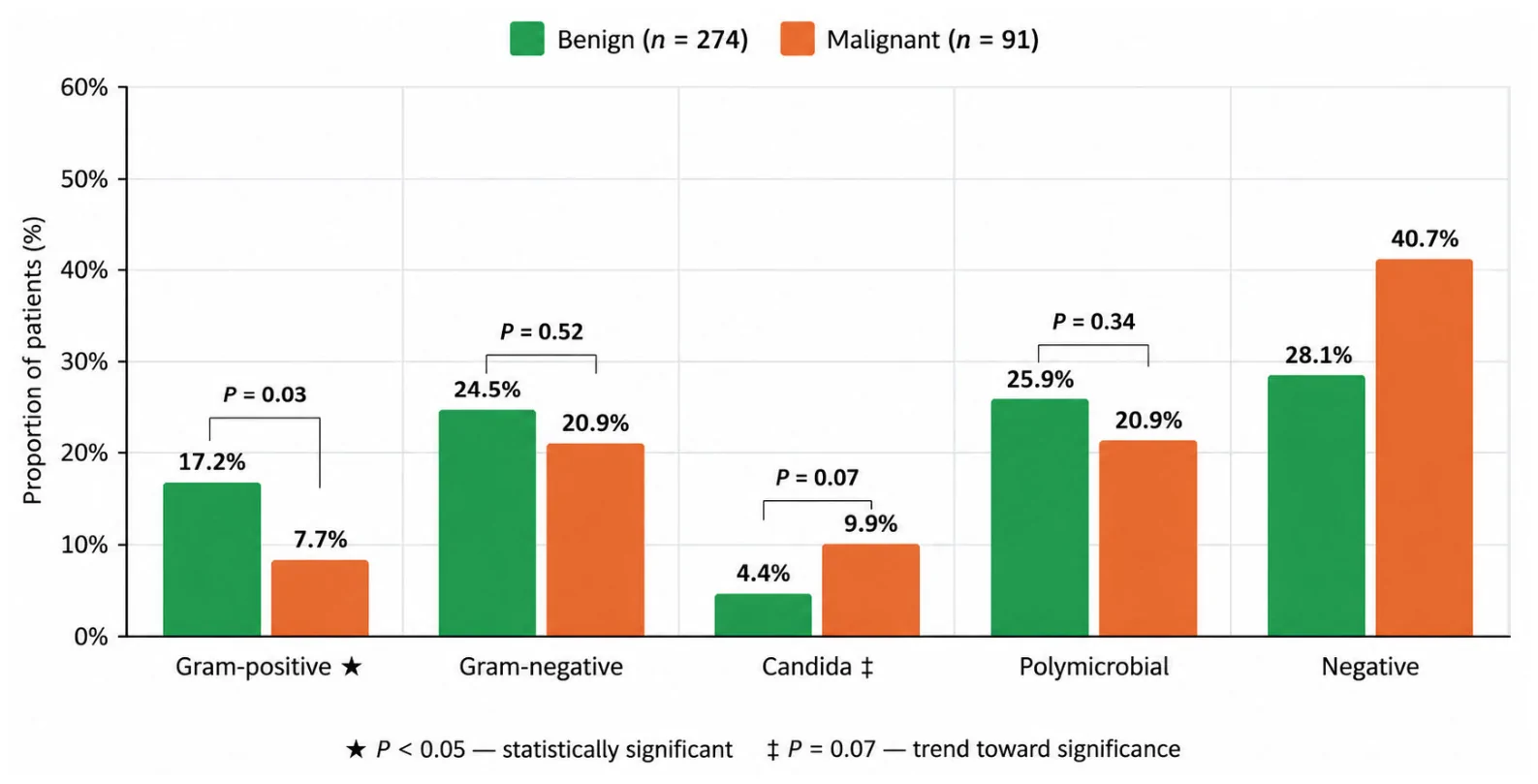
Bile culture distribution in benign versus malignant biliary obstruction. Gram-positive organisms were significantly more common in benign than in malignant obstruction (17.2% vs. 7.7%, *p* = 0.03). Gram-negative organisms were isolated at a similar proportion in both groups (24.5% vs. 20.9%, *p* = 0.52). *Candida* species were more frequently isolated in malignant obstruction, although this difference did not reach statistical significance (9.9% vs. 4.4%, *p* = 0.07). Polymicrobial growth occurred at a similar rate in both groups (25.9% vs. 20.9%, *p* = 0.34), while culture-negative results were more frequent in the malignant group (40.7% vs. 28.1%).

**Figure 3:**
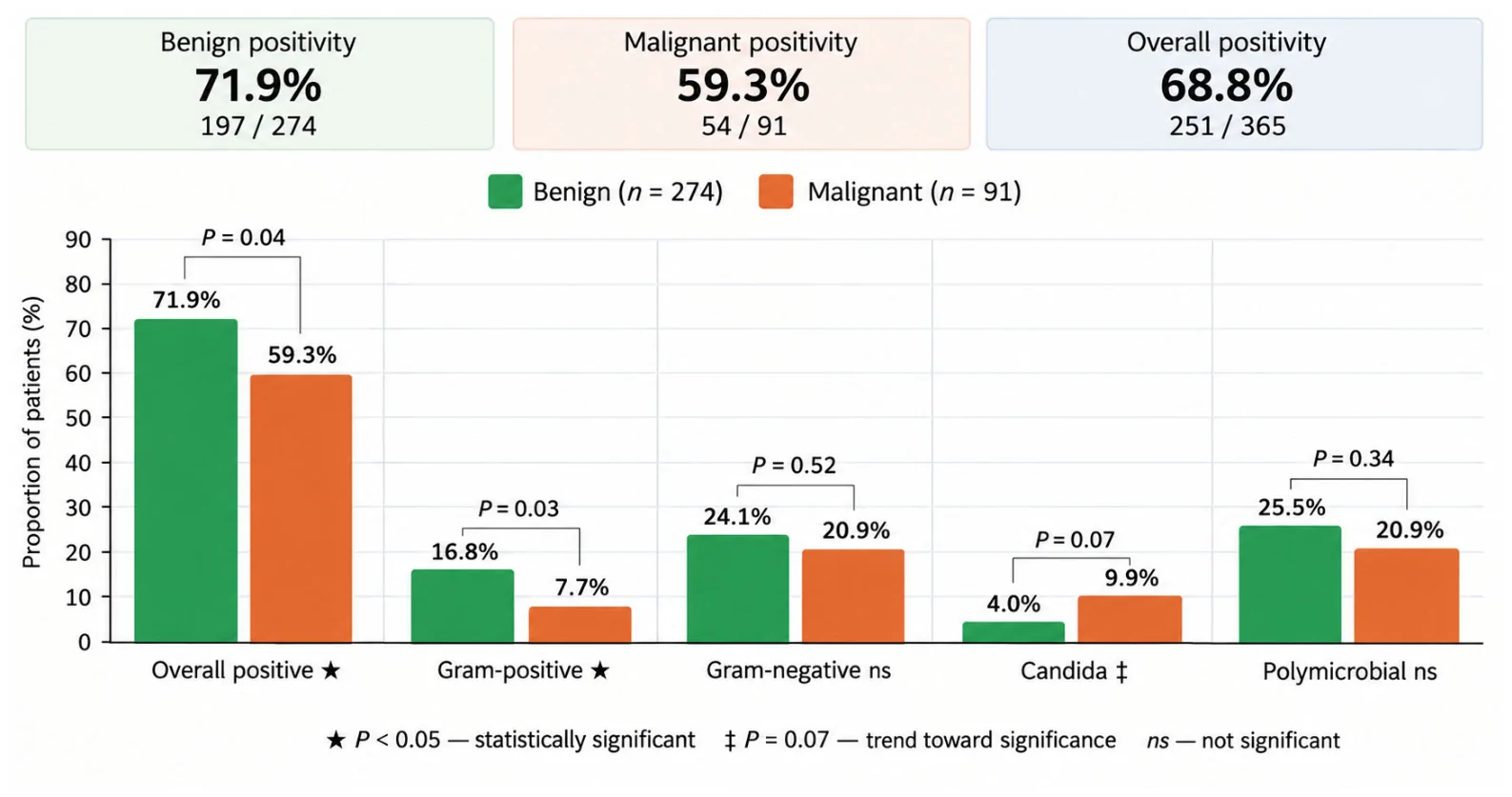
Microbial spectrum in benign versus malignant biliary obstruction: primary comparison. Overall bile culture positivity was significantly higher in benign than in malignant obstruction (71.9% [197/274] vs. 59.3% [54/91], *p* = 0.04). Among culture-positive samples, Gram-positive organisms were significantly enriched in benign obstruction (17.2% vs. 7.7%, *p* = 0.03). Gram-negative organisms did not differ significantly between groups (24.5% vs. 20.9%, *p* = 0.52); *Candida* species showed a non-significant trend toward higher prevalence in malignant obstruction (9.9% vs. 4.4%, *p* = 0.07); and polymicrobial growth was similar between groups (25.9% vs. 20.9%, *p* = 0.34).

**Table 1:** Baseline characteristics of the study population, stratified by benign versus malignant biliary obstruction.

Variable	Total (*n* = 404)	Benign Obstruction (*n* = 304)	Malignant Obstruction (*n* = 100)	Risk Difference (95% CI)	*p* Value
Age, years, median (IQR)	66 (55.8–74)	65.5 (54.8–75)	66 (57–73)	—	0.78
Sex, female, *n* (%)	182 (45%)	134 (44%)	48 (48%)	−4.0% (−15.2% to 7.3%	0.54
Diabetes mellitus, *n* (%)	99 (25%)	74 (24%)	25 (25%)	−0.8% (−10.4% to 9.1%)	0.87
Alkaline phosphatase, U/L, median (IQR)	180 (115.8–318.3)	145.5 (101–240.3)	386.5 (235–532.8)	—	<0.001
Total bilirubin, mg/dL, median (IQR)	2.6 (1.18–5.66)	2.0 (0.90–4.36)	6.97 (3.55–11.46)	—	<0.001
Direct bilirubin, mg/dL, median (IQR)	1.62 (0.52–3.98)	1.14 (0.41–2.98)	5.19 (2.31–8.18)	—	<0.001
AST (GOT), U/L, median (IQR)	104.5 (43.8–245)	96.5 (37–257.5)	119 (73.8–212.3)	—	0.27
ALT (GPT), U/L, median (IQR)	132 (44–284.8)	140 (38.8–290)	118 (60.5–257)	—	0.36
CRP, mg/dL, median (IQR)	1.69 (0.40–6.69)	1.39 (0.34–6.74)	2.13 (1.05–6.20)	—	0.15

Abbreviations: IQR, interquartile range; CI, confidence interval; AST, aspartate aminotransferase; ALT, alanine aminotransferase; CRP, C-reactive protein. Continuous variables are presented as median (IQR) and compared using the Mann–Whitney U test; categorical variables are presented as *n* (%) and compared using the chi-square test or Fisher’s exact test, as appropriate. SMDs for continuous variables were calculated as Cohen’s d based on means and standard deviations estimated from the reported medians and IQRs (SD ≈ IQR/1.35).

**Table 2:** Indications for cholangiography and the corresponding bile and blood sampling numbers.

Diagnosis	Patients (*n* = 404)	Bile Cultures (*n* = 365)	Blood Cultures (*n* = 270)
Benign bile duct obstruction	304 (75.2%)	274 (75.1%)	200 (74.1%)
Choledocholithiasis	265 (65.6%)	239 (65.5%)	180 (66.7%)
Surgical injury	8 (2.0%)	8 (2.2%)	5 (1.9%)
Pancreatic pseudocyst	2 (0.5%)	2 (0.5%)	0 (0%)
Pancreatic IgG4-related disease	5 (1.2%)	5 (1.4%)	2 (0.7%)
Primary sclerosing cholangitis	1 (0.2%)	1 (0.3%)	0 (0%)
Others (benign)	23 (5.7%)	19 (5.2%)	13 (4.8%)
Malignant bile duct obstruction	100 (24.8%)	91 (24.9%)	70 (25.9%)
Pancreatic cancer	47 (11.6%)	44 (12.1%)	32 (11.9%)
Cholangiocarcinoma	17 (4.2%)	16 (4.4%)	11 (4.1%)
Hepatocellular carcinoma	13 (3.2%)	12 (3.3%)	9 (3.3%)
Hepatic metastasis	6 (1.5%)	6 (1.6%)	4 (1.5%)
Others (malignant)	17 (4.2%)	13 (3.6%)	14 (5.2%)

Percentages are calculated using the total number of enrolled patients (*n* = 404), patients with an attempted bile culture (*n* = 365), and patients with a concurrently drawn blood culture (*n* = 270), respectively. The denominators therefore differ among the three columns.

**Table 3:** Comparison of culture yields and microbial diversity between benign and malignant obstruction.

Variable	Benign Obstruction (*n* = 274)	Malignant Obstruction (*n* = 91)	Risk Difference (95% CI)	*p* Value
Bile culture performed, *n* (%)	274 (90%)	91 (91%)	−0.8% (−7.4% to 5.7%)	0.81
Positive bile culture, *n* (%)	197 (71.9%)	54 (59.3%)	12.6% (1.1% to 24.0%)	0.04
Polymicrobial, *n* (%)	71 (25.9%)	19 (20.9%)	5.0% (−4.8% to 14.9%)	0.34
Monomicrobial Gram-positive, *n* (%)	47 (17.2%)	7 (7.7%)	9.5% (2.4% to 16.5%)	0.03
Monomicrobial Gram-negative, *n* (%)	67 (24.5%)	19 (20.9%)	3.6% (−6.2% to 13.4%)	0.52
Monomicrobial *Candida*, *n* (%)	12 (4.4%)	9 (9.9%)	−5.5% (−12.1% to 1.1%)	0.07

Categorical variables were compared using the chi-square test or Fisher’s exact test, as appropriate. Risk differences are presented with 95% confidence intervals (CIs). CI, confidence interval.

**Table 4:** Distribution of organisms identified in positive bile cultures (*n* = 339 isolates).

Organism	No. (%)
Gram-positive bacteria	96 (28.3%)
*Enterococcus* species	63 (18.6%)
*Streptococcus* species	15 (4.4%)
*Staphylococcus* species	11 (3.2%)
Others	7 (2.1%)
Gram-negative bacteria	211 (62.2%)
*Escherichia coli*	65 (19.2%)
*Klebsiella* species	45 (13.3%)
*Enterobacter cloacae*	24 (7.1%)
*Pseudomonas* species	22 (6.5%)
*Citrobacter* species	5 (1.5%)
Others	50 (14.7%)
*Candida*	32 (9.4%)
*Candida albicans*	29 (8.6%)
Other *Candida* species	3 (0.9%)
Anaerobes	27 (7.9%)

## Data Availability

The data presented in this study are available on reasonable request from the corresponding author, subject to institutional and ethical restrictions governing patient privacy.

## Abbreviations

The following abbreviations are used in this manuscript:

ERCP	endoscopic retrograde cholangiopancreatography
PTCD	percutaneous transhepatic cholangiodrainage
ASGE	American Society for Gastrointestinal Endoscopy
IDSA	Infectious Diseases Society of America
IQR	interquartile range
SMD	standardized mean difference
CI	confidence interval
CRP	C-reactive protein
AST	aspartate aminotransferase
ALT	alanine aminotransferase
ALP	alkaline phosphatase
MDRO	multidrug-resistant organism
IRB	Institutional Review Board
